# The mutual impacts of stem cells and sleep: opportunities for improved stem cell therapy

**DOI:** 10.1186/s13287-025-04235-3

**Published:** 2025-03-29

**Authors:** Sharif Moradi, Masoumeh Nouri, Mohammad-Taher Moradi, Reza Khodarahmi, Morteza Zarrabi, Habibolah Khazaie

**Affiliations:** 1 Department of Stem Cells and Developmental Biology, Cell Science Research Center, Royan Institute for Stem Cell Biology and Technology, ACECR, Tehran, Iran; 2 R&D Department, Royan Stem Cell Technology Co, Tehran, Iran; 3 Sleep Disorders Research Center, Kermanshah University of Medical Sciences, Kermanshah, Iran; 4 Medical Biology Research Center, Health Technology Institute, Kermanshah University of Medical Sciences, Kermanshah, Iran; 5 Department of Regenerative Medicine, Cell Science Research Center, Royan Institute for Stem Cell Biology and Technology, ACECR, Tehran, Iran

**Keywords:** Sleep medicine, Engraftment, Stem cells, Circadian clock, Self-renewal, Clock genes

## Abstract

Sleep is an indispensable physiological function regulated by circadian rhythms, which influence the biological pathways and overall health of the body. Sleep is crucial for the maintenance and restoration of bodily systems, and disturbances can lead to various sleep disorders, which can impair both mental and physical health. Treatment options for these disorders encompass lifestyle modifications, psychotherapy, medications, and therapies such as light therapy and surgery. Not only sleep deprivation has a significant impact on essential organs, but it also influences various types of stem cells in the body. In this review, we explore the connection between sleep and various types of stem cells, highlighting how circadian rhythms regulate stem cell activities that are vital for tissue regeneration and homeostasis. Disruptions in sleep can hinder stem cell self-renewal, homing, proliferation, function, and differentiation, thereby affecting tissue regeneration and overall health. We also discuss how transplantation of stem cells and their products may help improve sleep disorders, how sleep quality affects stem cell behavior, and the implications for stem cell therapies. Notably, while certain stem cell transplantations can disrupt sleep, enhancing sleep quality may improve the efficacy of these therapies. Finally, stem cells can be utilized to model sleep disorders, offering valuable insights into their underlying mechanisms.

## Introduction

Sleep is a distinctive and vital physiological function endowed with restorative strength and orchestrated by circadian rhythms [1]. It impacts the core biological operations of the human body, leading to the upkeep, restoration, and construction of bodily systems in harmony with internal and external cues [2]. Sleep timing is regulated by the central circadian clock and peripheral oscillators that function in response to the cycle of light and darkness [3, 4]. The biological functions of a cell are influenced by the circadian clock, which is the main determinant of the quality and quantity of our sleep. Not only sleep critically contributes to the functioning of all organs and tissues of the body, but also sleep disruption leaves a considerable negative impact on these functions, thereby adversely affecting the quality of life.

The major types of sleep disorders include, but not limited to, insomnia, sleep-related breathing disorders, central disorders of hypersomnolence, circadian rhythm sleep-wake disorder, parasomnias, and sleep-related mental disorders. These disorders can influence not only the patients’ mental health but also their physical health, highlighting the need to develop effective treatment for such people. The currently available treatments for sleep disorders include (i) a healthy sleep hygiene regime through changing sleeping routine, (ii) psychotherapy through cognitive behavioral therapy, (iii) medications, (iv) controlling sleep apnea through a ‘continuous positive airway pressure’ machine, (v) implanted neurostimulators, (vi) surgery, and (vii) light therapy.

Sleep deprivation or disruption leaves significant effects on the vital organs of the body. The brain suffers the most from sleep deprivation, which leads to cognitive impairment, irritability, memory lapses/loss, decreased creativity, impaired moral judgment, and enhanced stress. Sleep disruption also adversely affects the heart through increases in heart rate variability and an enhanced risk of strokes and cardiovascular diseases. Muscles are also influenced. They exhibit reduced accuracy, prolonged reaction time, aches, and tremors during sleep deprivation. Finally, the digestive system and its associated immune system are negatively influenced, leading to impairments in immune responses in the digestive system. These findings indicate that sleep disorders target both mental and physical aspects of a person’s normal life.

Sleep and sleep disruptions appear to have big effects on the functionality of stem cells. Stem cells are unique cells inside various tissues of the human body, which indispensably contribute to the regulation of tissue homeostasis. Not only these cells can self-renew under certain conditions in vivo but they can also differentiate into one or more types of differentiated cells in response to various developmental and/or environmental cues. Certain types of stem cells are integral to embryonic development and contribute to sustaining an organism’s homeostasis over its lifetime [5]. In terms of developmental potency, stem cells are categorized into unipotent, multipotent, and pluripotent stem cells. Unipotent stem cells are stem cells that can generate only one differentiated cell type upon exit from their stemness program. Examples of unipotent stem cells include spermatogonial stem cells, which can give rise to sperm, and satellite cells, which can differentiate into muscle cells. Multipotent stem cells are self-renewing cells capable of differentiating into several cell types within a single cell lineage. For example, hematopoietic stem cells (HSCs) exhibit long-term self-renewal and can produce all the various cell types in the blood cell lineage, e.g., white blood cells and red blood cells. Other examples include mesenchymal stem cells (MSCs), neural stem cells (NSCs), and intestinal stem cells (ISCs). Finally, pluripotent stem cells (PSCs) are immortal cells, meaning that they exhibit unlimited self-renewal, and have the ability to differentiate into all the cell types of the body as well as germ cells. Embryonic stem cells (ESCs), induced pluripotent stem cells (iPSCs), and germline pluripotent stem cells (gPSCs) are the major types of PSCs. Due to their multi-lineage differentiation potential, PSCs hold great potential in treating various types of diseases that are currently incurable. Notably, the unique features of stem cells stem from their unique gene regulatory networks which in turn determine their behaviors and activities.

The circadian rhythm helps to ensure that stem cell activities, such as tissue repair and renewal, are optimally timed with the body’s needs and environmental cues. This synchronization is crucial for preserving tissue integrity and promoting tissue regeneration, thereby maintaining the organism’s overall health [6]. When normal sleep and circadian rhythms are disrupted, the self-renewal ability and developmental potential of stem cells are clearly disrupted. Such aberrations in normal stem cell signaling and functioning lead to abnormalities in tissue renewal and regeneration, which in turn can adversely affect the overall homeostasis of the human body. This review aims to elucidate these complex interactions between sleep and stem cell dynamics, highlighting opportunities for improved therapeutic strategies. After providing a brief overview on sleep-wake disorders, we explore how sleep and sleep disorders affect stem cells. In particular, we discuss how the behavior of hematopoietic stem cells (HSCs) is influenced by sleep quality. HSCs have received particular attention in this study due to the extensive body of research examining their role in the context of sleep. This focus is largely due to the crucial role HSCs play in the immune system and the significant impact of sleep on immune function. Moreover, among the types of circulating stem cells in the blood, HSCs are generally the most abundant and are therefore more affected by the blood-borne hormones, e.g. melatonin and cortisol, that regulate sleep and circadian rhythms.

Sleep disturbances can exacerbate inflammation and induce changes in the bone marrow microenvironment, consequently affecting both the function and diversity of HSCs (discussed later in the article). Here, we explore how stem cell transplantation might enhance sleep disorders. Stem cell therapies are rapidly growing for treating various types of diseases, and it is important to realize that HSC transplantation (HSCT) impairs patients’ sleep. Interestingly, while some stem cell transplants can disrupt sleep, improving sleep quality may boost the efficacy of these treatments. On the other hand, impaired sleep reduces the success rate of stem cell treatments, highlighting a mutual impact between these two areas of medicine. Interestingly, transplantation of MSCs has been documented to improve sleep problems in animals. Moreover, we describe how stem cells can be used to model sleep disorders in a dish. Finally, we offer suggestions on how improving sleep quality may lead to a more successful stem cell therapy.

## An overview of sleep medicine and sleep disorders

Sleep medicine is a multi-disciplinary field that explores the complexities of sleep and its disorders. The primary goal of sleep medicine is to improve the quality of life for people with diverse types of sleep disorders. With growing awareness of the crucial role sleep plays in physical and mental health, and cognitive function, sleep medicine has emerged as a vital area for both research and clinical practice. Sleep disorders are not mere inconveniences, but can disrupt daily life and lead to serious health problems like cardiovascular disease, obesity, and mental health conditions [7]. Sleep disorders are a wide spectrum of conditions that adversely affect normal sleep patterns. Here are a few of the most prevalent sleep disorders:

### Insomnia

Insomnia is defined by frequent dissatisfaction with staying asleep, falling asleep, waking too early, or an inability to return to sleep, which can result in distress or impairment in a patient’s daily life. Insomnia is often associated with anxiety, depression, and other psychological factors. The prevalence of insomnia has risen dramatically in recent years, emphasizing the need for effective treatment strategies [8, 9].

### Sleep-related breathing disorders

Sleep-related breathing disorders are a group of conditions marked by abnormal breathing patterns during sleep. They are categorized into several main types, including sleep apnea, sleep-related hypoventilation disorders, and sleep-related hypoxemia disorders [10].

Sleep apnea is characterized by repeated interruptions in breathing during sleep. There are two main types of sleep apnea: obstructive sleep apnea, which happens when throat muscles relax and block the airway, and central sleep apnea, which occurs when the brain does not send the right signals to the breathing muscles. Sleep apnea is frequently linked to obesity and other metabolic disorders and leads to significant health complications, including hypertension, cardiovascular disease, and cognitive impairment [11–14].

### Central disorders of hypersomnolence

Central disorders of hypersomnolence are characterized by excessive daytime sleepiness (EDS) that persists despite adequate nighttime sleep [15, 16]. These disorders are distinct from other sleep disorders, as they do not arise from disrupted nocturnal sleep, sleep apnea, or circadian rhythm misalignment. The primary disorders within this category include narcolepsy (types 1 and 2), idiopathic hypersomnia, and Kleine-Levin syndrome [16]. Narcolepsy is a neurological condition marked by EDS and frequent, uncontrollable sleep attacks. This condition disrupts the brain’s ability to control sleep patterns, leading to fragmented sleep and symptoms like sleep paralysis, cataplexy, and hallucinations when falling asleep. There are two types of narcolepsy: narcolepsy type 1 involves a significant loss of neurons that produce the neurotransmitter orexin (hypocretin), which is involved in regulating sleep and wakefulness. Narcolepsy type 2 does not involve cataplexy but still includes excessive daytime sleepiness [17, 18].

### Parasomnias

Parasomnias represent a category of sleep disorders characterized by abnormal movements, behaviors, emotions, perceptions, and dreams that occur during the processes of falling asleep, while sleeping, between sleep stages, or during arousal from sleep. Common types of parasomnias include sleepwalking, night terrors, confusional arousal, sleep eating, sleep sex, teeth grinding (bruxism), somniloquy (sleep talking), rhythmic movement disorder, and restless legs syndrome (RLS) [19]. RLS results in an irresistible need to move the legs, typically accompanied by unpleasant feelings such as tingling, itching, or crawling [20, 21], leading to difficulty falling asleep and disrupted sleep patterns. RLS has been linked to genetic factors and certain medical conditions, including iron deficiency [22], kidney failure [23], and pregnancy [24].

### Circadian rhythm sleep-wake disorders

Circadian rhythm disorders arise when there is a mismatch or disruption between an individual’s internal biological clock and their external environment. This can manifest in various ways, such as delayed sleep phase disorder or shift work disorder, ultimately affecting overall health and well-being. These disorders can lead to chronic sleep deprivation and have been linked to a range of health problems [25, 26].

The consequences of sleep disorders extend far beyond nighttime disturbances, significantly affecting physical health. Chronic sleep deprivation increases the risk of obesity, cardiovascular disease, diabetes, and weakens the immune system. Additionally, sleep disorders can exacerbate mental health conditions such as anxiety and depression, creating a vicious cycle that further disrupts sleep quality [27]. Inadequate sleep also leads to cognitive consequences, including impaired memory, reduced attention span, and diminished problem-solving abilities. This can negatively affect academic performance and workplace productivity. Furthermore, irritability and mood swings caused by poor sleep can also strain social relationships [28]. These multifaceted impacts underscore the critical need for effective treatments in sleep medicine to break this cycle and improve overall well-being.

## Effects of sleep and sleep disruption on stem cells

### The impact of sleep and circadian rhythms on bodily organs and tissues

The growing understanding of sleep disorders and the impact of modern life on human health have introduced the concept of circadian medicine. This field emphasizes the importance of aligning human activities with circadian rhythms to maintain optimal tissue and organ function [29]. In humans, the relationship between the incidence of certain cancers and cardiovascular diseases in occupations that disrupt normal sleep patterns has been reported [30, 31]. Studies have also revealed a correlation among fragmented sleep, elevated levels of norepinephrine in the heart tissue, and an increased incidence of cardiovascular diseases in mice via accumulation of intracellular copper, which in turn results in increased myocardial cuproptosis and apoptosis [32]. Additionally, there is evidence of higher rates of diabetes, cancer, and cardiovascular diseases in mouse models lacking a molecular clock [29, 33], highlighting the role of sleep and its downstream physiological processes in tissue homeostasis and repair, in addition to its time-keeping function. Processes affected by sleep disruption, such as tissue homeostasis, gene regulation, DNA repair, and immune system responses, are all potential tumor promoters [34]. The relationship between tumor suppressor genes and clock genes at the molecular level further evidences the impact of sleep disruption on oncogenesis [35]. In fact, disruption of the circadian rhythm is considered a contributing factor in tumor progression because circadian molecular feedback loops are involved in various aspects of tumor growth and progression, such as the cell cycle, repair mechanisms, and apoptosis [34].

**Fig. 1:**
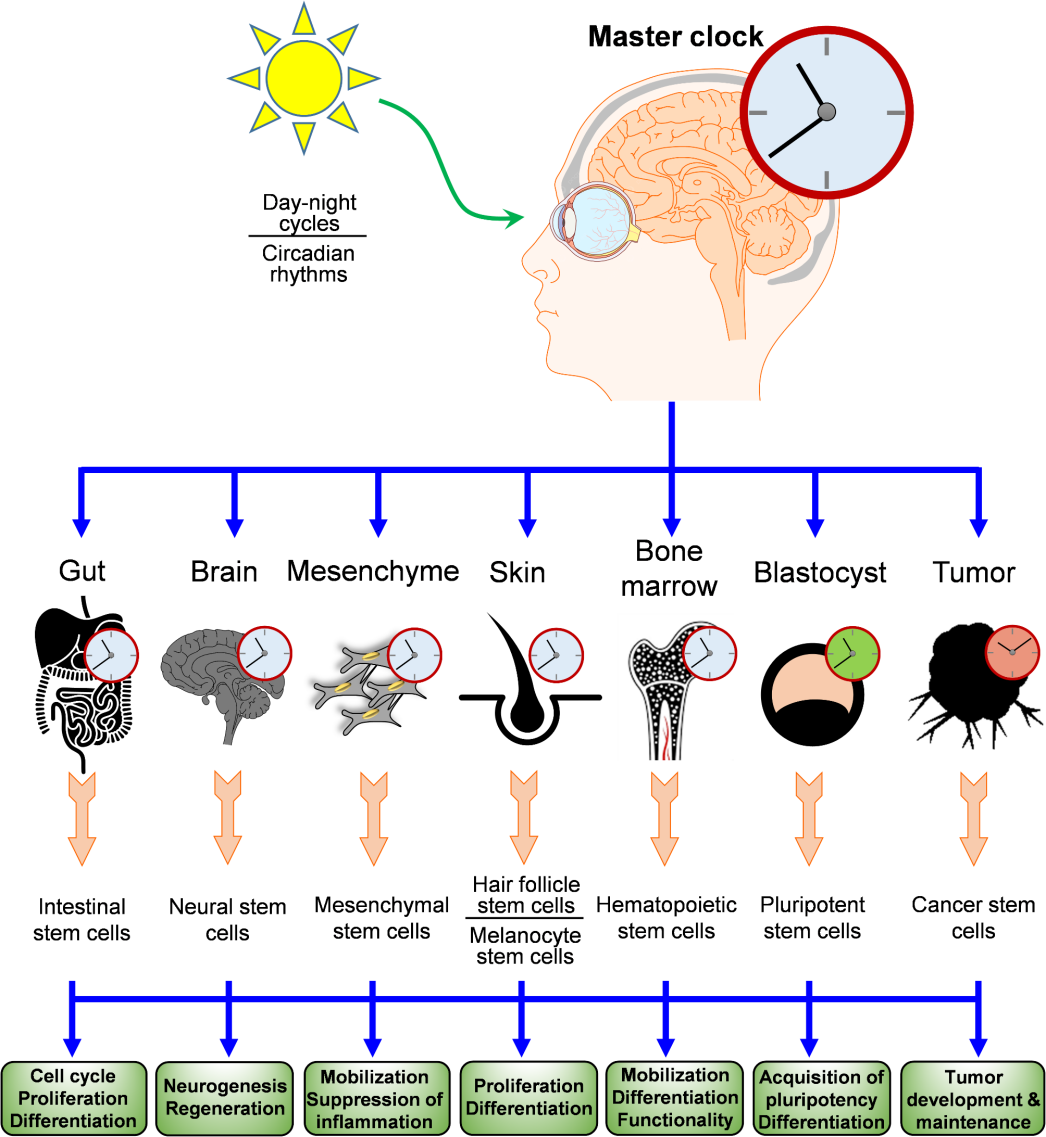
The impact of circadian rhythms on various types of stem cells. In the hippocampus, the clock genes control the neurogenesis of neural stem cells. Circadian rhythms also regulate the proliferation and differentiation of hair follicle stem cells and melanocyte stem cells in the skin. In the digestive tract, the cell cycling of intestinal stem cells is under the control of the light and dark cycles. The proliferation, differentiation, and mobilization of HSCs also depend on the circadian clock. Circadian clock also contributes to the regulation of PSCs, although their response to circadian rhythms depends on their differentiation status. When undifferentiated, PSCs show less rhythmicity in clock gene expression; however, they activate rhythmic expression of several clock gene upon differentiation. The circadian clock in cancer stem cells is often dysfunctional or functions in favor of tumorigenesis. In general, circadian clock genes regulate the signaling pathways, cell cycle, differentiation and fate of embryonic and adult stem cells

Circadian rhythms and sleep quality affect human physiology through hormones such as cortisol and melatonin, cytokines, and clock gene expression. Cumulative evidence highlights the effect of circadian rhythms on the regenerative abilities of various cell types and stem cells particularly in the skin, intestinal lining, blood (hematopoietic) system, and brain [36] (Fig. 1). This effect is evident in their capacity in promoting wound healing, significant tissue replacement, and continuous replenishment [37].

### The impact of sleep and circadian rhythms on MSCs

Evidence suggests that circadian rhythms affect the fate of MSCs through multiple mechanisms. These include the hormone secretion pathway, the production of clock gene products that influence the expression and activity of transcription factors, and the induction of epigenetic modifications [38]. Circadian rhythms have been reported to induce daily oscillations in the expression of the *CXCL12* gene in MSCs [39]. Therefore, it can be hypothesized that regulating sleep may influence the outcomes of MSC transplantation.

Melatonin, which is a key sleep-regulating hormone primarily secreted by the pineal gland, has been shown to play a critical role in MSC-based regenerative medicine. It enhances the viability and therapeutic effects of MSCs by regulating reactive oxygen species (ROS) generation and immune responses [40]. In human studies, melatonin supplementation has been associated with improved MSC survival and function, suggesting its potential as a therapeutic agent in regenerative medicine [40, 41]. Melatonin possesses the ability to directly bind to ROS and reactive nitrogen species. It not only enhances the activity of antioxidant enzymes but also inhibits pro-oxidant enzymes. Additionally, melatonin acts as an antioxidant by chelating transition metals, thereby promoting MSC survival and inhibiting oxidative stress in these cells [42].

Circadian rhythms, regulated by melatonin and cortisol, have a significant impact on MSC function. Disruptions in circadian rhythms can adversely affect MSC behavior. Human studies have shown that maintaining regular sleep patterns can enhance MSC proliferation and differentiation, highlighting the importance of circadian regulation in MSC biology. The findings from human studies suggest that optimizing sleep patterns and hormonal regulation can enhance the therapeutic potential of MSCs [41, 43–45].

MSCs have been observed to be mobilized from the bone marrow into the bloodstream in response to hypoxia and inflammation [46]. Recurrent intermittent hypoxia and inflammation are two features that are observed in patients with obstructive sleep apnea, suggesting that MSCs are induced to mobilize into the bloodstream in these patients. In fact, sera from the rat models of obstructive sleep apnea was reported to stimulate the motility of MSCs [47]. This finding suggests that obstructive sleep apnea might induce the secretion of certain chemotactic proteins into the bloodstream, thereby promoting MSC mobilization.

### Effects of sleep and circadian rhythms on the behavior of HSCs

Not only body organs are under circadian control, but also individual cells are carefully regulated by their respective internal biological clocks. Interestingly, it has been estimated that over 40% of human proteins in at least one body organ are under circadian control, suggesting that all ingredients of an organism’s body are influenced by circadian rhythms [37, 48, 49]. In this regard, the nervous system sends specific circadian information via adrenergic signals to connect the central biological clock in the suprachiasmatic nucleus to the bone marrow microenvironment, thereby controlling clock gene expression in bone marrow stromal cells and drive circadian oscillations during the migration of HSCs [50]. During the nighttime, inhibitory cholinergic signals decrease the nocturnal egress of bone marrow HSCs. At the same time, cholinergic and adrenergic signaling pathways collaborate to promote the recruitment of HSCs to the bone marrow during their active phase (i.e. daytime for humans and nighttime for rodents) [50]. Research on HSC-sleep relationship has been much more extensive than on other stem cells due to HSCs’ critical role in the immune system and sleep’s significant impact on immune function. Sleep disruptions can alter the bone marrow environment, affecting HSC function and diversity. Importantly, HSCs were the first stem cells identified and rigorously defined for therapeutic use, discovered by Ernest McCulloch and James Till in the 1960s. While other types of stem cells such as ESCs were identified later, HSCs were the pioneers in stem cell therapy. Below, we start our discussion on the impact of sleep on HSCs by highlighting how HSCs are regulated by circadian rhythms and external cues.

Since oscillations in oxygen levels can control the function of HSCs and synchronize molecular clocks, it appears that HSC regulation requires interplay between hypoxia signaling and molecular clock machinery [39]. Furthermore, the constitutive trafficking patterns of HSCs in the bloodstream to various tissues indicate circadian oscillations in homing to and egress from the bone marrow niche [51]. Mobilization of HSCs is regulated by circadian fluctuations originating from the CXCL12-CXCR4 axis, with daily CXCL12 gene expression oscillations observed in bone marrow MSCs (reviewed in [39]). Overall, these findings suggest that circadian rhythms and hypoxia may jointly promote dynamic chromatin changes to trigger transcriptional events that regulate the normal physiological functions of HSCs.

It has been over half a century since the initial findings were published on the influence of diurnal cycles on the proliferation of bone marrow cells, which are composed of a diverse array of various cell types [52]. A rare population of bone marrow-resident cells, known as HSCs, provides the lifelong production of blood- and immune cells in the body to support healthy hematopoiesis [53]. The continuous regeneration of the blood system by bone marrow-derived HSCs is critically dependent on the tightly-regulated processes of quiescence, self-renewal, and differentiation, which are fundamental characteristics of HSCs. This intricate regulation, spanning from molecular level to environmental cues, is essential to support the turnover of 90% of the body’s cellular mass each day, which is composed of nearly 284 billion blood cells [54]. At the heart of this massive cellular turnover are HSCs, which initiate and sustain this continuous process.

In darkness, HSCs engage in self-renewal processes to maintain their reservoir, which is mediated by the secretion of the darkness hormone melatonin. Conversely, daily onset of light triggers the activation of proliferation and differentiation in HSCs by the release of epinephrine within the bone marrow, as shown by the effects of light and dark cycles on HSCs using animal models and controlled light/dark cycle manipulation [55, 56]. Therefore, the self-renewal and differentiation of HSCs are clearly under the careful control of the day-night cycle through the activity of the body’s circadian clock.

A large population-based study has demonstrated that inadequate sleep quality affects HSC differentiation, leading to an augmentation of myeloid cell production and a higher risk of developing atherosclerosis [57]. This phenomenon is inversely associated with leukopoiesis, as evidenced in murine models [58]. McAlpine et al. showed in animal models and human subjects that fragmented sleep can durably influence HSC function by altering epigenetic programming, enabled through limited accessibility at enhancers important for lymphocyte lineage differentiation and increased accessibility at enhancers associated with myeloid lineage differentiation. Consequently, this was shown to result in a bias towards monocyte differentiation. Additionally, it reduces hematopoietic clonal diversity and HSC proliferation, ultimately changing the immune system function [59]. The effects of sleep on HSC differentiation are not yet well understood, and further research is needed to elucidate more details of sleep influences on this fundamental process in HSCs. These findings highlight the crucial role of sleep in the differentiation of HSCs and underscore its importance in public health.

Investigations into the interplay between sleep and hematopoiesis have elucidated a neuro-immune axis that governs the modulation of immune and cardiovascular functions through the regulation of hematopoietic diversity in mice [58]. These findings indicate that inadequate and erratic sleep patterns, which is prevalent in contemporary lifestyles, significantly contribute to the predisposition and incidence of immunological disorders and tumors in humans [60, 61]. Experimental studies have shown that a wide array of immune parameters is affected by a sleep-immune crosstalk. Immune system activation by microbial challenges can trigger an inflammatory response, affecting sleep duration and quality. Conversely, sleep enhances immune defense by promoting the production of immune cells and cytokines, while poor sleep can lead to chronic inflammation associated with diseases such as diabetes, atherosclerosis, and neurodegeneration [62]. This intricate relationship underscores the importance of sleep for maintaining immune homeostasis.

### The effects of sleep and circadian rhythms on PSCs and cancer stem cells

Circadian rhythms play a crucial role in regulating the behavior of PSCs [63, 64]. Interestingly, the response of PSCs to circadian rhythms depends on their differentiation status. In this regard, the circadian transcriptional-translational feedback loop is absent in PSCs as well as germ cells [5, 65], whose behavior is primarily influenced by pluripotency genes through negative feedback loops [66]. This characteristic is also observed in ESCs in vitro [67]. Despite the fact that clock gene-associated circadian rhythms are missing in mouse and human PSCs, key clock genes such as *PER1*, *PER2*, *CRY1*, and *CRY2*, *CLOCK*, and *BMAL1* are actively expressed in these cells [68]. Recent evidence has shown that the circadian clock gene *CRY1* is a regulator of somatic cell reprogramming and iPSC production [69]. Clock genes in iPSCs do not respond to synchronizing agents but exhibit a unique circadian-like rhythm driven by temperature changes, likely regulated through HIF-1α rather than clock genes [70]. In cancer stem cells, as a subpopulation of bulk tumors, the circadian clock is often dysfunctional or acts in favor of the development and maintenance of tumors [71]. Therefore, the evidence indicate that circadian rhythms have a direct effect on the proliferation, division, differentiation, and migration of tissue stem cells (Fig. 1). Hence, the quality of sleep is a major factor in maintaining tissue homeostasis and the regeneration of the body through the activity of stem cells residing in each tissue. Additionally, the significant co-occurrence of certain diseases with sleep disruption may also induce changes in the regulatory patterns of tissue stem cells.

### The impact of sleep and circadian rhythms on other types of stem cells

Circadian rhythms also regulate other types of stem cells. Neurosphere cultures from the mouse subventricular zone (SVZ) revealed that a circadian clock activates 3–4 days after differentiation begins, suggesting that it is inactive in the NSC state [72]. Neurogenesis of NSCs in the hippocampus is regulated by clock genes such as *BMAL1*,* CRY*, and *PER2* [73]. The daily rhythmic release of norepinephrine (NE) from LC NEergic neurons was reported to regulate the circadian variation of NSC activation through β3-adrenoceptors. NE/β3-adrenoceptors-mediated signaling controls NSC activation through the molecular clock component BMAL1 [74]. Disruptions in circadian rhythms result in an increased number of M-phase NSCs, their excessive activation, and the subsequent depletion of the NSC pool. This further substantiates the impact of sleep on these cells, and this disruption can adversely affect associated brain functions, including learning and memory [74].

Proliferation and differentiation of hair follicle stem cells and melanocyte stem cells, that are respectively responsible for hair growth and skin pigmentation, have also been found to be regulated by the circadian clock [75]. Research suggests a link between sleep disorders and hair loss. Disrupted sleep can elevate cortisol levels, harming hair follicles and potentially causing conditions such as telogen effluvium. Reduced melatonin from poor sleep also negatively affects hair growth. Additionally, sleep deprivation impacts stem cell activity involved in hair regeneration, further contributing to hair loss [76, 77], suggesting that improving sleep quality may help mitigate these effects and support healthy hair growth. ISCs, with their high and continuous regenerative capacity, are among other tissue stem cells whose cell cycling is affected by circadian rhythm [78, 79]. Circadian rhythms play a crucial role in regulating the behavior of ISCs. These rhythms control various cellular processes, including ISC proliferation, differentiation, and self-renewal. It has been reported that ISC activity peaks at specific times of the day, aligning with the body’s internal clock. Disruptions in circadian rhythms, such as those caused by irregular sleep patterns, can impair ISC function and potentially affect the homeostasis and overall function of the gastrointestinal tract [79, 80]. Molecularly, circadian rhythms regulate ISC behavior feedback loops involving core clock genes such as *BMAL1* and *PER*. Key signaling pathways, including Wnt, Notch, and Hippo, were found to be affected by circadian rhythms and play important roles in ISC regulation [79, 80].

All of these vital phenomena have been found to be regulated through the rhythmic patterns of transcription, post-transcriptional modifications, and translation mechanisms [81]. These rhythms align with body regenerative and reparative patterns, emphasizing the crucial role of tissue stem cells [82]. Cellular processes such as cell division, differentiation, migration, self-renewal, homeostasis, and tissue repair are controlled by circadian rhythms. On the other hand, clock genes affect the cell cycle, signaling pathways, and ultimately the differentiation and fate of adult stem cells [83]. The proliferation and gene expression patterns of stem cells in the intestine, skin, nervous system, and blood are dependent on light and dark cycles [75]. These findings indicate that circadian rhythms, and therefore sleep quality, play a crucial role in the external regulation of these processes.

Aligning the timing of stem cell transplantation, including HSCT, with the recipient’s circadian rhythm is crucial [84, 85]. Disregarding this alignment may lead to complications such as differentiation impairment, immune rejection, and graft-versus-host disease (GVHD) [38, 84–86]. It has been reported that the synchronization of the circadian rhythms of clock genes in transplanted MSCs with the host organism is of crucial importance for the successful transplantation and integration of MSCs in type 2 diabetes mellitus [87]. In conclusion, proper timing can enhance the engraftment success and overall effectiveness of the transplantation.

## Impact of HSCT on sleep: disruptions in sleep and related implications

The transplantation of various types of stem cells for stem cell-based therapies holds great promise in replacing and/or regenerating diseases or lost tissues. HSCT remains a cornerstone therapy for various hematologic disorders and malignancies. HSCT can treat a variety of diseases, including blood cancers (e.g. leukemia, lymphoma, and multiple myeloma), autoimmune diseases (e.g. lupus and multiple sclerosis), bone marrow failure syndromes (e.g. severe aplastic anemia and sickle cell disease), immune deficiencies including severe combined immunodeficiency (SCID), and certain metabolic disorders such as Gaucher disease and Hurler syndrome [88]. Despite its long-standing use, the determinants of transplant success are not fully elucidated. However, considering the significant influence of circadian rhythms as well as optimizing the timing of HSC harvesting and transplantation based on circadian biological effects may improve transplantation outcomes [51, 89].

Despite its significant therapeutic applications, HSCT has serious adverse effects. The intensive nature of HSCT, which includes a harsh conditioning regimen involving high-dose chemotherapy and/or radiation therapy, as well as the hospital environment, frequent monitoring, and prolonged hospital stays, can cause significant side effects and discomfort. These factors negatively impact patients’ overall well-being.

Evidence suggests a significant decrease in the sleep quality of patients after receiving an HSCT, with this decline being observable long after the transplant [90]. More severe and long-term sleep disorders have been experienced in younger patients receiving allogeneic transplants [90, 91]. It is now clear that sleep disruption has a negative effect on the biological functions of HSCs under normal conditions. Therefore, in order to increase the homing and engraftment chances of HSCs, it is recommended to adjust and provide suitable sleeping conditions for patients prior to (stem) cell transplantation. Regulating sleep patterns in both donors and recipients before and after transplantation may improve the success rates of engraftment [92]. For example, light exposure is known to enhance the migration of HSCs and the development of mature cells via increasing norepinephrine levels. Conversely, darkness elevates melatonin levels, promoting HSC homing and self-renewal under physiological conditions [55, 93]. These results indicate that regulating light-dark cycles and circadian rhythms may be beneficial in patients receiving HSCT [92].

The success rate of HSCT has been shown to decrease to 50% in recipient mice whose donors experienced sleep disruption. This observation was found to be due to the downregulation of the microRNA (miR)-19 and the consequent reduction in the homing and migration of transplanted HSCs. MicroRNAs are small, regulatory non-coding RNAs that control gene expression at the post-transcriptional level, thereby modulating virtually all biological pathways and developmental processes [94–96]. In fact, is has been documented that external factors such as sleep deprivation affect miR-19, decreasing its expression levels and subsequently upregulating its direct gene targets particularly *suppressor of cytokine signaling (SOCS)*. This indicates that environmental conditions can modulate the molecular pathways involving miR-19, thereby impacting HSC migration and homing capabilities following transplantation [97]. These findings indicate that the success rate of HSCT could be significantly improved if sleep problems associated with the harsh procedure of HSCT are prevented or minimized. Therefore, it would be worthwhile to further investigate sleep treatment modalities to determine if they can enhance the efficiency of HSCT.

## Modeling sleep and sleep disorders using stem cells

Stem cells offer a powerful tool for creating disease models in a dish. In particular, PSCs such as ESCs and iPSCs are of critical importance for disease modeling due to their unique characteristics. These characteristics include long-term self-renewal, which makes these cells immortal, and multi-lineage differentiation potential, which enables them to produce all the diverse cell types of the body [98, 99]. Uncovering the mechanisms underlying sleep disturbances is important for the development of effective treatments, but this is challenging due to the lack of relevant, easy-to-use disease models. ESCs and iPSCs can overcome the limitations of animal models for disease modeling. These cells can be easily obtained and propagated in vitro, serving as an immense cell source for various biomedical applications including disease modeling [100]. Patient-specific iPSCs and PSC-differentiated neurons have become increasingly popular as in vitro models for sleep and circadian rhythm disorders. Due to their huge differentiation potential, PSCs enable the development of relevant experimental models capable of recapitulating various neuroendocrine pathways and complex neural circuits, thereby allowing for the identification of novel therapeutic targets for the effective treatment of sleep disorders.

Various differentiation protocols are available to derive neuronal cells from PSCs for modeling sleep disorders [101–103]. These protocols typically replicate the normal developmental pathways that occur over the course of neural specification and determination. To obtain PSC-derived neurons, PSCs are first induced by modulation of several signaling and cellular pathways to become neural cells. Next, the resulting neural cells are subjected to neuronal differentiation via stimulation of various biological processes driving the differentiation of neurons. Neuronal differentiation consists of neurogenesis and synaptogenesis, which together give rise to the generation of mature PSC-derived neurons [103].

Yokoi et al. investigated the effects of several sleep-wake-associated neuromodulators on human iPSC-derived neurons and found that the neurotransmitters (i.e. serotonin, acetylcholine, histamine, orexin, and noradrenaline) promoted a clear increase in synchronized burst firings (SBFs) in human iPSC-derived dopaminergic neurons in a dose-dependent manner. Stimulation of PSC-derived glutamatergic neurons with electrical 1 Hz was observed to decrease SBFs. Their results suggest that cyclic neuromodulator administration can mimic sleep–wake states and indicate that long-term depression-like phenomena could be induced by low-frequency stimulation in human iPSC-derived neurons. These observations are similar to sleep-related changes in brain activity, which demonstrates that PSCs provide a great opportunity for modeling sleep [104]. The comparison of the activity of different types of PSC-derived neurons indicated that dopaminergic neurons differentiated from human iPSCs elicited lower rates of firings and SBFs compared to motor neurons on microelectrode arrays [104]. Due to their ability to be used in pathological contexts, human PSCs were also differentiated to generate neurons capable of displaying epileptiform SBFs, which were suppressed using anti-epilepsy drugs [104].

Kaneko et al. examined how clock genes respond to synchronizing agents, such as forskolin and dexamethasone, in human undifferentiated and differentiated iPSCs, and found that the expression of clock genes was not altered by synchronizing stimulation in undifferentiated iPSCs, which may be, at least partly, due to the low levels of BMAL1 as the core clock protein. However, a unique circadian-like rhythm of the clock genes was induced by the temperature rhythm [70]. Interestingly, the unresponsiveness of undifferentiated iPSCs to synchronizing agents resembles to a feature of these cells to hide disease phenotypes when iPSCs are disease-specifically derived from patients [105]. Once the cells are differentiated, the disease characteristics are revealed and, in the case of circadian rhythms, the clock genes start responding to synchronizing stimulations.

Three-dimensional PSC cultures such as cell aggregates, organs-on-chip, and tissue explants offer a more reliable opportunity to model sleep disorders, as traditional 2D cultures of PSCs fail to replicate some aspects of normal sleep and its disorders and are suboptimal in enabling the investigation of cell-cell- and cell-ECM interactions as well as electrophysiological networks involved in controlling sleep [106]. In this regard, several 3D scaffolds such as hydrogels have been used to culture human PSC-derived brain organoid systems allowing for microelectrode array-based recordings of electrophysiological network activity [107–109]. Human PSC-derived organoids enable to model human brain activity, which is because they can self-assemble into 3D tissue-like structures composed of neuronal and glial cell types [110–112]. In fact, the rate of firing in organoids was observed to be significantly higher compared to 2D cultures [113].

Human PSCs have recently been used to model neuronal circadian rhythms. Using human iPSC-derived neural progenitor cells and cortex-like glutamatergic neurons, it was observed that dysregulation of the molecular clock adversely influences neural progenitor cells. The circadian rhythms were found to be more resistant to treatments and external entrainment factors [114]. Interestingly, while spontaneous differentiation of ESCs results in the gradual activation of the circadian clock in these cells, pluripotent reprogramming (also known as iPSC reprogramming) reduces the rhythmicity of the clock genes’ expression. Notably, the activation of the circadian clock upon directed differentiation of ESCs has been found to promote oscillatory expression of several clock genes. These findings show that the activation or inactivation of the circadian clock is a reversible process that is tightly connected with the developmental state of the cells (reviewed in [64]).

In vitro differentiation of PSCs is a great tool to effectively investigate the specialized neuronal cells that are directly involved in sleep regulation. In this regard, hypocretin (HCRT) and melanin-concentrating hormone (MCH) are known to be brain neuropeptides exclusively synthesized by the lateral hypothalamus that are critically implicated in various functions, including sleep. For example, lack of HCRT or its receptors has been reported to lead to narcolepsy along with cataplexy in animal models and humans. Therefore, in vitro generation of the specialized neurons secreting these neuropeptides (known as HCRT- or MCH neurons) from PSCs would provide an invaluable in vitro cell-based tool for sleep modeling. Recently, Seifinejad et al. reported the successful establishment of an optimized differentiation protocol for the conversion of iPSCs into HCRT and MCH neurons [115]. These iPSC-derived neurons would be of significant importance for the interrogation of mechanisms underlying sleep disorders. Therefore, PSC-based modeling and mechanistic study of sleep disorders may pave the way for the discovery of new drugs and/or treatment modalities for sleep disorders.

## Treating sleep disorders using stem cells and their products

Not only stem cells have been used for modeling sleep and circadian rhythms, they also appear to be effective in treatment of sleep disorders upon transplantation. Obstructive sleep apnea is an underestimated sleep disorder, which may result in damages in multiple organs, including injuries in the lungs. It is characterized by recurrent intermittent hypoxia during sleep and inflammatory responses, which lead to pathological manifestations typical of obstructive sleep apnea. It has been observed that MSCs are mobilized from the bone marrow into the bloodstream in response to these stimuli, i.e. hypoxia and inflammation [46]. This observation is also the case for several other stem cells [39, 116], suggesting that these cells might help ameliorate the negative effects of the above-mentioned events. There are numerous studies indicating that MSCs serve anti-inflammatory functions upon transplantation [117–122]. Interestingly, Carreras and colleagues reported that MSCs played protective, anti-inflammatory roles upon injection into a rat model of obstructive sleep apnea [46].

Another study conducted with the same rat model of obstructive sleep apnea reported that serum from the rat models enhanced the motility and regenerative potential of MSCs in vitro [47]. Analysis of the chemotaxis of these cells revealed that the serum from the apneic rats considerably enhanced the migration of the cells, suggesting that obstructive sleep apnea may drive the secretion of certain chemotactic proteins into the bloodstream, thereby mobilizing MSCs toward the affected tissues. Notably, the systemic injection of MSCs immediately before the induction of recurrent airway obstructions in rats dramatically decreased the systemic inflammation induced by apneas [47, 123].

Xu et al. used extracellular vesicles obtained from adipose-derived MSCs to examine if they could improve pathological manifestations of obstructive sleep apnea-hypopnea syndrome. They found that obstructive sleep apnea-hypopnea syndrome was efficiently alleviated in an animal model of the disease (i.e. a rat model of recurrent airway obstruction mimicking obstructive sleep apnea) upon injection of the extracellular vesicles, which was accompanied by a significant decrease in inflammation, oxidative stress, and lung injury. Mechanistically, the extracellular vesicles were revealed to shuttle a specific microRNA called miR-22-3p from adipose-derived MSCs into pneumonocytes, which subsequently downregulated KDM6B and HMGA2 in the target cells, leading to the attenuation of apoptosis, oxidative stress, and inflammation in the animal model [124]. These results indicate that MSCs exert their anti-inflammatory effects, at least partly, through their secretions and paracrine effects, which is consistent with previous reports [125–127].

Lin et al. sought to determine whether extracellular vesicles secreted by human umbilical cord MSCs exert any therapeutic impact in mice with sleep deprivation. Treatment with the extracellular vesicles was found to ameliorate cognitive impairment and anxiety-like behaviors observed in sleep-deprived mice through regulating synaptic function and inhibiting inflammation (by downregulating TLR4 and p65) in the hippocampus of the mice [128]. Therefore, MSCs play a protective role by counterbalancing the main challenges of sleep disorders. Table 1 provides a more detailed, molecular description of the mutual interactions between sleep and stem cells.

**Table 1:** Interventional studies on the mutual interaction of sleep and stem cells

Stem cell type	Intervention	Species	Study design	Main results	Mechanism	Refs
HSCs	Sleep disruption	Mouse; Human	In vivo	Increases HSC proliferation; reduces clonal diversity; promotes myeloid differentiation and monocytosis; primes cells for exaggerated inflammatory responses	Enhanced genetic drift; epigenetic reprogramming via HAT and HDAC activity	[59]
		Mouse	In vivo	Promotes atherosclerosis; leads to more Ly-6C^high^ monocytes; increases monocytosis; decreases circulating monocytes	Reduced hypocretin production; increased CSF1 generation	[58]
	HSCT	Human	*Longitudinal observational study*	Significantly reduces sleep quality; induces depression and anxiety; promotes inflammation	Higher circulating levels of IL-6; harsh HSCT procedure	[90]
NSCs	Circadian rhythm disruption (acute sleep deprivation)	Mouse	In vivo	Enhances LC NEergic neuronal activity; over-activates NSCs; depletes NSC pool	Increased norepinephrine release or β3-adrenoceptor signaling	[74]
ISCs	Photoperiod alteration	Drosophila	In vivo	Synchronizes intestinal cells; promotes stem cell circadian clock function	Modulation of Wnt, Hippo, and Notch pathways	[79]
MSCs	Recurrent airway obstruction	Rat; serum from rat	In vivo	Induces early MSC mobilization into bloodstream; enhances MSC regenerative potential	Anti-inflammatory secretome from MSCs; IL-1β reduction	[46, 47, 123]
	Recurrent airway obstruction	Rat	In vivo	Attenuates OSA-induced atrial fibrillation; reduces myocardial inflammation and fibrosis	Decreased IL-1β plasma levels; increased MMP-2 expression	[132]
	MSC-derived EV injection into OSA model	Mouse	In vivo	Ameliorates cognitive impairment and anxiety-like behaviors by regulating synaptic function and inhibiting inflammation	TLR4 and p65 downregulation	[128]
	MSC-derived EV injection into OSA model	Rat	In vivo	Reduces inflammation and oxidative stress; promotes lung tissue regeneration by decreasing lung injury	miR-22 shuttling from EVs into pneumonocytes; KDM6B and HMGA2 downregulation	[124]

HSCs: hematopoietic stem cells; HSCT: hematopoietic stem cell transplantation; HAT: histone acetyltransferase; HDAC: histone deacetylase; CRY1: circadian repressor cryptochrome 1; ISCs: intestinal stem cells; NSCs: neural stem cells; MSCs; mesenchymal stem cells; LC: locus coeruleus; NEergic: norepinephrinergic; EV: extracellular vesicle; OSA: obstructive sleep apnea

**Fig. 2:**
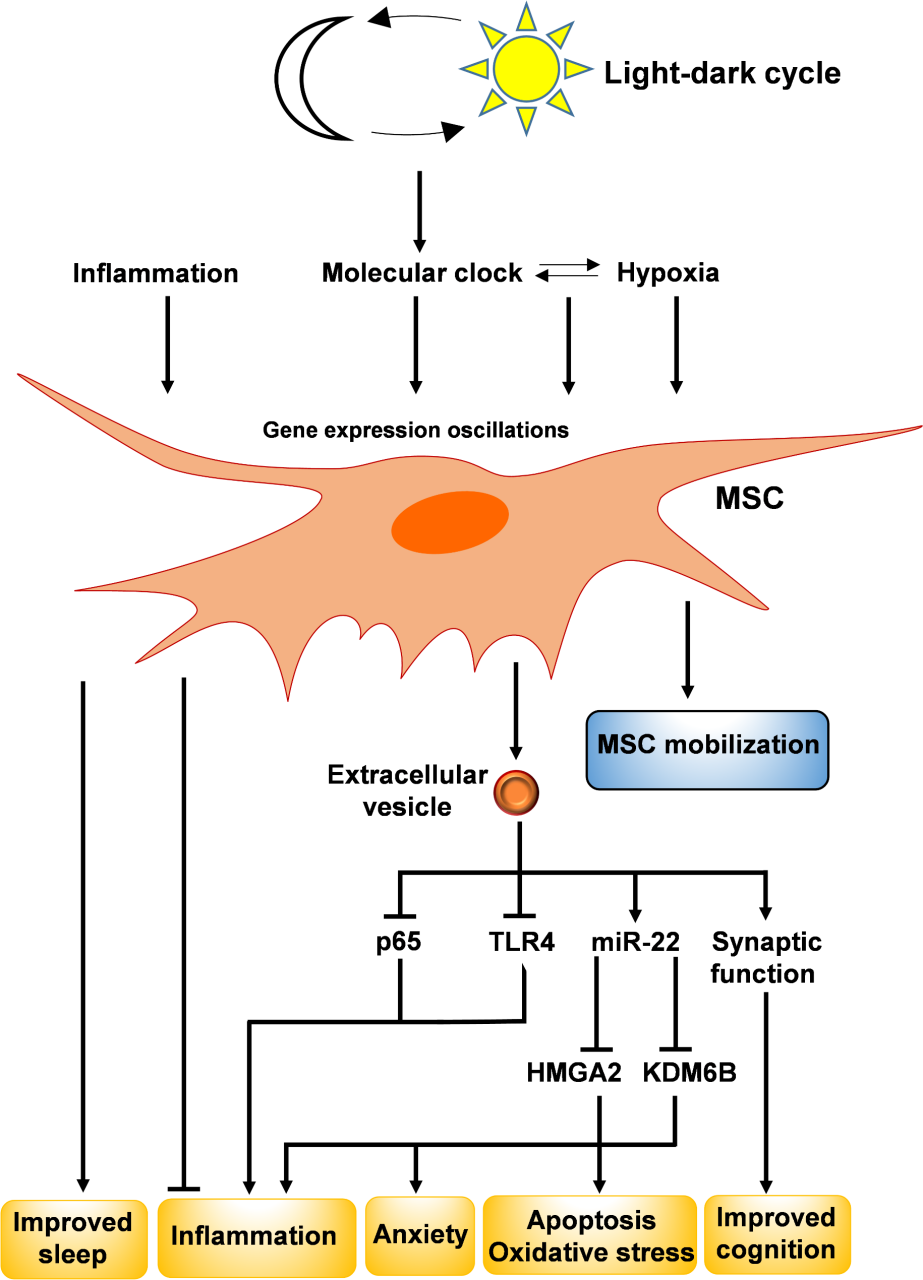
The mutual impacts of MSCs and sleep. The day-night cycles promote rhythmic oscillations in MSC gene expression. Moreover, extrinsic stimuli such as hypoxia and inflammation induce MSC mobilization into the bloodstream. On the other hand, MSCs play anti- protective, inflammatory roles during sleep disruption, which is partially exerted through MSC-derived extracellular vesicles. These extracellular vesicles decrease inflammation, oxidative stress, and lung injury in animal models of sleep disorders. They shuttle miR-22-3p, which suppresses KDM6B and HMGA2 in the cells, thereby inhibiting inflammation, apoptosis, and oxidative stress. Extracellular vesicle-mediated repression of TLR4 and p65 also leads to inhibition of inflammation, improvement of synaptic function, amelioration of cognitive impairment, thereby resulting in a decrease in anxiety-like behaviors in the sleep-deprived animals

It would be important to further characterize the secretory factors from MSCs that mediate the amelioration of inflammation associated with sleep disturbances. Identification of these factors may enable the development of new defined treatments based on MSCs or MSC-derived factors to be used for the safe and efficacious therapy of sleep disorders. The discovery of new molecular treatments based on MSC studies and/or using MSC-derived products, e.g. MSC-derived extracellular vesicles, instead of using MSC itself would be a safer next step, as the transplantation of whole cells for the treatment of sleep disorders may come up with specific challenges associated with cell therapy. Figure 2 summarizes how MSCs and MSC-derived extracellular vesicles play important roles in the improvement of sleep disruptions.

## Can improving sleep quality lead to a more successful stem cell therapy?

It is obvious that disruptions in sleep exert significant adverse effects on both mental and physical health of individuals. If left untreated, sleep disorders can lead to serious problems for the normal life of patients. There is a diverse array of diseases that are currently incurable; for some of them, stem cells hold enormous potential as cell-replacement therapies. The therapeutic potential of stem cells arises from their two unique features, i.e. long-term self-renewal and developmental potency that enable them to generate various therapeutically relevant cells for transplantation.

**Fig. 3:**
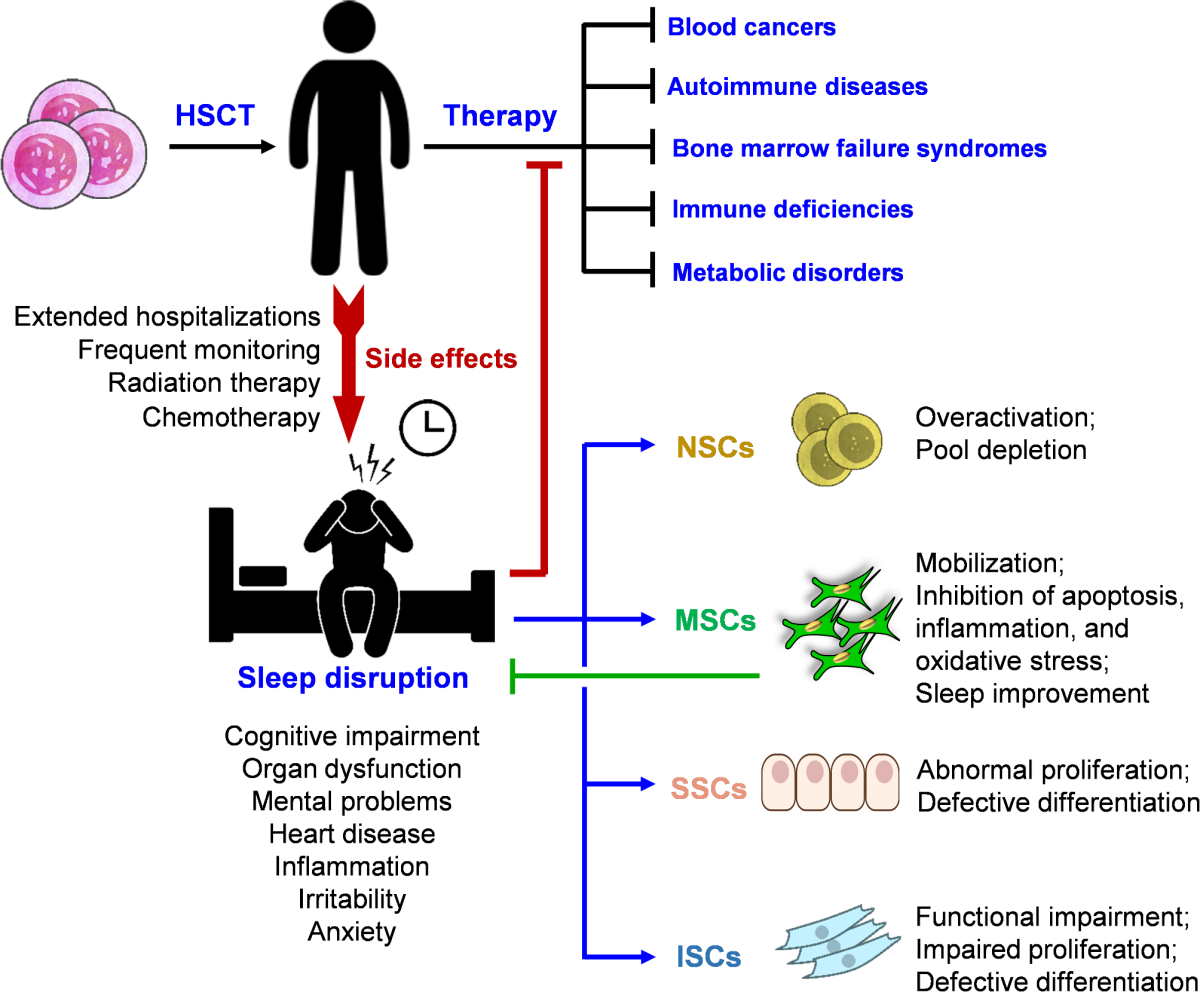
Mutual impacts of sleep and stem cells. HSCT is utilized to treat various blood diseases, including blood cancers, immune deficiencies, and autoimmune diseases. However, HSCT is associated with significant adverse effects in patients. One of the notable side effects is sleep disruption, which adversely affects the proliferation, differentiation, and functions of stem cells. Unlike other stem cell types, MSCs are activated and mobilized into the bloodstream in response to sleep disturbances. Subsequently, MSCs secrete factors, including various cytokines, to exert anti-inflammatory effects on sleep impairment. The molecular mechanisms of these interactions are summarized in Table 1

It has been shown in sleep-deprived animals that stem cell transplantation is less efficient than in animals with normal sleep pattern. Similar results have been obtained when sleep-deprived human subjects received stem cell transplantation. These findings clearly show that it would be of crucial importance to make sure that the stem cell recipients have a regular and healthy sleep pattern before stem cell therapy, otherwise the transplanted stem cells meet with failure upon homing, function, diversity, and/or differentiating, as reported previously [59, 129]. To treat sleep disorders in such patients, it would preferable to use therapeutic strategies that are minimally invasive yet yield significant therapeutic efficacy. As a viable therapeutic option, it seems that stem cell transplantation itself has a potential in improving sleep problems. The transplantation of MSCs or MSC-derived extracellular vesicles has been found to ameliorate symptoms associated with sleep disruption in animals [47, 124]. Figure 3 illustrates the mutual interaction of sleep and stem cells.

As the most common type of clinical stem cell therapy, HSCT is being practiced in various stem cell clinics worldwide. The transplantation of HSCs has been documented to cause sleep disruption in stem cell recipients [90, 130, 131]. Given that (i) stem cell transplantation itself may cause sleep problems and (ii) sleep problems reduce the efficacy of stem cell therapies, it would be critically important to devise strategies that enable efficacious stem cell treatment without causing sleep disturbances. The first simple step toward addressing this issue is supportive psychotherapy. Additionally, short-term sleep medications or supplements might be prescribed before stem cell transplantation. However, it should be ensured that those pre-treatments do not impair or interfere with the efficiency of stem cell therapy. Long-term use should be avoided due to risks of abuse and dependence, as well as potential negative impacts on stem cell functionality. Further research would be required to determine how successful stem cell therapy could be achieved without inducing sleep problems.

## Concluding remarks

In the present review article, we discussed the critical role of sleep as a physiological function regulated by circadian rhythms, which affects the body’s biological operations and overall health. Sleep is essential for the maintenance and restoration of bodily systems. Sleep deprivation significantly affects vital organs such as brain, heart, muscles, and the digestive system. We highlighted the relationship between sleep and stem cells, noting that circadian rhythms help regulate stem cell activities crucial for tissue repair and homeostasis. Disruptions in sleep can impair stem cell function, affecting tissue regeneration and overall health. The review also explored the interplay between sleep and stem cells, particularly how sleep quality influences HSCs and the implications for stem cell therapies. Extracellular vesicles derived from MSCs appear to improve pathological manifestations of sleep apnea. Stem cell therapies can be physically and psychologically demanding for patients. The procedures and recovery often disrupt sleep patterns. On the other hand, sleep helps maintain the microenvironment that supports stem cell function and homing. By improving sleep, patients can bolster their overall health, which in turn enhances the efficacy of stem cell therapies and promotes better outcomes.

HSCT involves an intensive conditioning regimen, comprising high-dose chemotherapy and/or radiation therapy to eradicate existing bone marrow, followed by the infusion of healthy HSCs. This rigorous process is associated with significant side effects including major sleep disturbances that can persist for months post-transplantation. Consequently, HSCT is considered more arduous compared to MSC transplantation. The MSC transplantation involves the isolation of MSCs from sources such as fat or skin, their expansion in the laboratory, and subsequent reintroduction into the body via injections. This procedure is generally less intensive and has fewer side effects compared to HSCT. Future research should further explore these connections to establish effective interventions that address both sleep disorders and stem cell therapy outcomes. Moreover, multi-omics approaches should be utilized to enhance our understanding of the mechanisms of sleep-stem cell interactions in general, and sleep-MSC interactions in particular, offering comprehensive insights into their epigenetic and transcriptomic interactions. Finally, stem cells can be used to model sleep disorders, providing insights into their mechanisms.

## Data Availability

Not applicable.
